# Is the blood of a surgeon performing HIPEC contaminated by irinotecan, its major metabolites and platinum compounds?

**DOI:** 10.1515/pp-2020-0141

**Published:** 2021-03-03

**Authors:** Guillaume Saint-Lorant, Simon Rodier, Jean-Marc Guilloit, Sophie Ndaw, Mathieu Melczer, Stéphanie Lagadu, Agnès Palix, Raphaël Delépée

**Affiliations:** Normandie Univ, UNICAEN, ABTE, Caen, France; Department of Pharmacy, CHU de Caen, Caen, France; Department of Surgery, Comprehensive Cancer Center F. Baclesse, Caen, France; Toxicology and Biomonitoring Department, INRS, Vandoeuvre, France; Department of Occupational Health, Comprehensive Cancer Center F. Baclesse, Caen, France; Comprehensive Cancer Center F. Baclesse, Caen, France

**Keywords:** antineoplastic drugs, blood contamination, HIPEC

## Abstract

**Objectives:**

Hyperthermic intraperitoneal chemotherapy (HIPEC) is a beneficial surgical technique for patients, but the surgeons are being exposed to cytotoxic drugs. Few biomonitoring studies were led on blood samples in the context of HIPEC. This study aimed to evaluate the surgeon’s plasmatic and red blood cell (RBC) contamination by irinotecan, two of its major metabolites and platinum compounds.

**Methods:**

HIPEC procedures performed using the coliseum techniques were observed between September 2015 and April 2018 in a French comprehensive cancer center. Irinotecan and its metabolites SN-38 and APC were dosed by UHPLC with a limit of quantification determined at 50 pg/mL. Platinum compounds were dosed by inductively coupled plasma mass spectrometry with a limit of quantification determined at 16 pg/mL.

**Results:**

Despite collective and personal protective equipment, 80% of plasma samples were contaminated by irinotecan and 33% by platinum compounds out of 21. The results showed that the surgeon was contaminated after HIPEC and even after a period of HIPEC inactivity. Nineteen percent of plasmatic samples and 45% of RBC samples were contaminated by SN-38, the active metabolite of irinotecan. APC was only found in some RBC samples (33%).

**Conclusions:**

Even if this study shows blood contamination by irinotecan, two of its major metabolites (including active SN-38) and platinum compounds both in the plasma and RBC of a surgeon performing the HIPEC procedures, further studies should be performed to confirm these results. Additional studies should be carried out to further investigate the contamination in the context of HIPEC and more broadly in the hospital.

## Introduction

Hyperthermic intraperitoneal chemotherapy (HIPEC) began to be performed in the United States during 1980s and in Japan and France [1], [2], [3]. The focus of this technique is metastasis carcinomatosis which is caused by macroscopic primary malignancies such as mesothelioma, pseudomyxoma and peritoneal secondary tumors, mainly from colorectal cancer. HIPEC is still being evaluated for use with the secondary origin of gastric or ovarian tumors [4], [5]. This surgery can be performed using either closed-abdomen or open-abdomen HIPEC techniques. The latter is called the “coliseum technique” as described by Sugarbaker [6]. This most common technique allows an improvement of cytotoxic biodistribution and is also preferred by surgeons because the anastomosis is performed after HIPEC [7], [8], [9]. The incidence of patients eligible for Cytoreductive surgery and HIPEC is estimated to be 29,000 to 41,000 per year in the United States [10]. Drugs commonly used are platinum complexes, mitomycin C (MMC), docetaxel and irinotecan, but the best drug to use for HIPEC is still unknown today [11], [12], [13]. Irinotecan appears to be metabolized to an active metabolite, SN-38 (300–1,000-fold more active than the parent), via carboxylesterase and to inactive metabolites, APC (7-ethyl-10-[4-N-(5-aminopentanoic acid)-1-piperidino] carbonyloxycamptothecin) and NPC (7-ethyl-10-[4-(1-piperidino)-1-amino] carbonyloxycamptothecin), via CYP 3A4. Irinotecan and its active metabolite, SN-38, inhibit the action of topoisomerase I, an enzyme that produces reversible single-strand breaks in DNA during DNA replication. These single-strand breaks relieve torsional strain and allow DNA replication to proceed. Irinotecan and SN-38 bind to the topoisomerase I-DNA complex and prevent religation of the DNA strand, resulting in double-strand DNA breakage and cell death. The precise contribution of SN-38 to the activity of irinotecan in humans is not known [14]. Irinotecan is cell cycle phase-specific (S-phase) [15].

Pharmacokinetic characteristics of oxaliplatin and its metabolites have been evaluated in both blood, plasma and ultrafiltrate [16], [17], [18], [19]. After administration, oxaliplatin rapidly forms three active metabolites: dichloro (DACH)-Pt, monochloro (DACH)-Pt and diaquo (DACH)-Pt. These metabolites can bind irreversibly to many blood or tissue components before being eliminated by the urinary tract, mainly as inactive conjugates. The rapid metabolism of oxaliplatin makes it necessary to assay all platinum complexes formed *in vivo* [20]. It is also necessary to take into account the free and bound forms of platinum. Platinum bound to plasma proteins and erythrocytes is pharmacologically inactive but represents the most important part of the dose found in the blood [19], [20]. The analysis of platinum (free and bound) must therefore be performed in plasma and erythrocytes for it to be a good biological indicator of exposure (IBE). The terminal elimination half-life of oxaliplatin for 300 h allows for post-exposure analysis of surgery. The terminal half-lives of the derivatives formed *in vivo*, estimated at approximately 240 h, also allows the analysis of cytotoxic blood levels at 20 h after HIPEC, in order to be certain that a systemic passage has taken place after inhalation or skin contamination [19].

Irinotecan was not mentioned in the International Agency for Research on Cancer (IARC) classification. According to the IARC classification, cisplatin was classified in group 2A (probable carcinogens). No other platinum derivatives were classified.

There is growing evidence that hospital care unit surfaces are contaminated by different antineoplastic drugs such as irinotecan, and platinum compounds [21], [22], [23]. Detectable levels of these drugs have still been found since 1979 in workers’ urine and imply occupational exposure [24], [25]. There are mainly two different biological pathways to validate healthcare workers’ contamination: urine and blood. Regarding using urine, there are different limitations: the metabolites’ stability, different volume, urine dilution and variable hydration levels of the surgeons from whom the urine is collected may not be suitable [26], [27]. Platinum compounds have been detected in blood samples of different healthcare workers [28]. However, there is no literature currently available about healthcare workers being contaminated by irinotecan, especially by its metabolites in the hospital and particularly during HIPEC.

The objective of this study was to evaluate the surgeon’s plasmatic and red blood cell contamination by irinotecan, two of its major metabolites and platinum compounds.

## Materials and methods

This study was conducted in one of the two regional comprehensive cancer centers in France. In this hospital, HIPEC was performed by the same single surgeon, who uses the coliseum technique. This study was a longitudinal prospective observational case study with repeated measures. The use of HIPEC was observed between September 2015 and April 2018. During this period, 17 HIPEC procedures were performed in 17 patients with the following etiologies: five cases of peritoneal pseudomyxoma; 10 cases of carcinomatosis of colorectal origin; one case of peritoneal mesothelioma; and one case of appendicular carcinomatosis. The median PCI score was 9 (2–26). The median number of resected organs was three (1–4). Nineteen blood samples were collected.

Usually, HIPEC was performed twice a month (every 15 days) in the same operating room using Sun chip apparatus, BF type, class 1 (Gamida, Eaubonne, France). The surgeon wears different personal protective equipment (PPE) before and after HIPEC and during HIPEC (Table 1). Before and after HIPEC, the surgeon wears non-latex sensitive gloves and a surgical gown. The first step of the HIPEC procedure is preceded by a surgical resection and maximal cytoreductive surgery CC-0 with residue less than 1 mm [29]. However, the surgery leaves the abdominal cavity and viscera with some microscopic residual disease, and systemic chemotherapy is generally not effective because of low drug penetration [30]. The technique then consists of administering cytotoxic drugs directly into the abdominal cavity. A bath of cytotoxic drugs diluted in solvent was made: dextrose 5% solution (2 L/m^2^) was injected into the abdominal cavity and heated to 43 ± 1 °C for a period of 30 min according to the protocol. During HIPEC, the surgeon wore PPE dedicated to HIPEC: PVC nitrile waterproof boots, a surgical gown with fabric reinforcement, a mask filtering facepiece particles (FFP category 3) and three pairs of gloves (one pair of latex gynecological gloves and two pairs of neoprene gloves). The three pairs of gloves are changed every 15 min. The surgeon manipulated the viscera to allow homogeneous penetration of the cytotoxic drugs into the abdominal cavity and between organs. This latter step allowed an improvement of cytotoxic biodistribution; however, it increased the risk of the surgeon being contaminated with irinotecan. A smoke evacuator light Evaculight (Daeshin, Seoul, South Korea) is also used during the surgery. The operating room temperature was monitored and kept between 20.9 and 24.1 °C; room pressure was kept between 993 and 1,038 mmHg.

**Table 1:** Surgeon’s personal protective equipment.

Name	Trade name	Reference	Manufacturer
**Before and after HIPEC**			
Non-latex sensitive gloves	Gammex latex free	340,007,060	Ansell, Richmond, Australia
disposable surgical gown	High Performance comfort single-use surgical gown	7693K	3M Maplewood, USA
**During HIPEC**			
PVC nitrile waterproof boots	AGRO 4000	02096	Sarraizienne, Celles sur Durolle, France
Latex gynecological gloves powder free 500 mm	Long cuff surgical gloves	114607P	Euromedis, Neuilly-sous Clermont, France
Neoprene gloves	Protexis	2D73DP70	Cardinal Waukegan, USA
Surgical gown with fabric reinforcement	Eclipse	9509CEA	Medline, Châteaubriant, France
Mask filtering facepiece particles (FFP category 3)	Gas/vapor and particulate respirator	4,279	3M, Maplewood, USA

Regarding cytotoxic drugs, in the operating room of the hospital, 5-FU was administered by IV route at a dose of 400 mg/m^2^ 1 h before HIPEC. Then, oxaliplatin (at a dose of 300 mg/m^2^) and irinotecan (at a dose of 200 mg/m^2^) were administered intraperitoneally during the study [11]. The half-lives of irinotecan and SN-38 are *T*_1/2_=14.2 and 13.8 h, respectively, for the terminal phase. These half-lives allowed samples to be collected the next morning after HIPEC. In case of cutaneous contamination, a delay in the pharmacokinetics must be considered because of the rate of absorption. Nevertheless, this delay was difficult to quantify. A coagulation citrate tube (BD Vacutainer 9NC, reference: 367,704, Plymouth, UK) was used for irinotecan and its metabolites and a clot activator tube (BD Vacutainer CAT, reference: 369,032, Plymouth, UK) was used for platinum compounds. The method of quantifying irinotecan and its two metabolites used total human blood, the French blood bank (Etablissement français du sang, Bois-Guillaume, Rouen, France) kindly supplied blood samples that were used as blanks for molecule quantification. This method shows the concentrations of the molecules in blanks. The blood samples were centrifuged to separate the plasma from the RBCs. For both plasma and RBCs, the first step consisted of adding topotecan as the internal standard (IS); this standard was used to improve the precision of quantification. The second step was to precipitate the proteins, and the supernatants were diluted in water to reach an acetonitrile content compatible with solid phase extraction (SPE). Irinotecan, its metabolites and the IS were then extracted from samples by an SPE procedure using Oasis HLB cartridges (Waters, Milford, MA, USA). After a drying step, the sample was dissolved in 20 µL of mobile phase before injection into a Nexera X2 UHPLC interfaced with an electrospray triple quadrupole mass spectrometer (LCMS-8030Plus; Shimadzu, Kyoto, Japan) used in the multiple reaction monitoring (MRM) acquisition mode after positive ionization. Two MRM transitions from the fragmentation of the [M+H]^+^ ion were recorded for each compound. The method validated for irinotecan and its metabolites assessment followed EMEA and FDA guidance criteria [31], [32].

Platinum determination was carried out with an 820-MS inductively coupled plasma mass spectrometer (ICP-MS, Bruker Daltonics, Champs-sur-Marne, France). A Scott spray chamber and a micromist nebulizer were used. To quantify platinum in blood samples, a matrix matched calibration curve was performed in 10-fold diluted urine with 2% v/v nitric acid. The calibration range was performed from 5 to 200 ng/L and all samples were analyzed in duplicate. The laboratory participated in the intercomparison programme 57/2016 (GEQUAS) and was certified for the determination of platinum.

The limit of quantification (LOQ) was determined at 50 pg/mL for irinotecan and the metabolites and 16 pg/mL for platinum.

The approval number received by the Committee for the Protection of Persons (CPP) concerning North-West III for this study was A16-D49-VOL.30.

## Results

Nineteen blood sample collections from the surgeon. The median duration of surgery was 420 min (300–600). Median patients’ blood losses were 200 mL (50–1,000). All blood samples were collected with a constant interval between the end of the HIPEC procedure and sampling (18.5 ± 1.78 h) respecting the pharmacokinetic characteristics of irinotecan and its metabolites and the platinum compounds.

Regarding plasma contamination by irinotecan and its metabolites, 15 out of 19 plasmatic samples were contaminated by irinotecan, corresponding to 79%. Irinotecan was quantified in 13 samples (corresponding to 68%) with a minimum of 92 pg/mL, a maximum of 266 pg/mL and a median of 100 pg/mL and was detected in two out of 19 samples, corresponding to 10%. SN-38 was detected in four out of 19 samples, corresponding to 21%. No APC was detected in the plasma (Table 2).

**Table 2:** HIPEC conditions, blood and RBC contamination of the surgeon to irinotecan, SN-38 and APC. <50 means that the compound was detected but below the limit of quantification (50 pg/mL).

Date of blood sampling	HIPEC the day before	Time between end of HIPEC and blood sample, hour	Time since the last HIPEC procedure, day	Oxaliplatin dosage administered during HIPEC, mg	Whole blood	Irinotecan dosage administered during HIPEC, mg	Plasma			RBC		
					^194^Pt, pg/mL		Irinotecan, pg/mL ± SD	SN-38, pg/mL	APC, pg/mL	Irinotecan, pg/mL ± SD	SN-38, pg/mL ± SD	APC, pg/mL
29/09/2015	Yes	20	14	531	<16	354	125 ± 19	--	--	--	--	<50
27/10/2015	Yes	17	28	558	--	372	125 ± 19	--	--	<50	--	50 ± 4
03/11/2015	Yes	17.5	7	609	--	406	100 ± 15	--	--	257 ± 46	--	--
10/11/2015	Yes	12.5	7	570	--	380	--	<50	--	<50	127 ± 17	<50
08/12/2015	No	NA	28	NA	<16	NA	103 ± 16	--	--	241 ± 54	<50	--
05/01/2016	Yes	18.5	56	567	<16	378	200 ± 30	<50	--	114 ± 34	<50	--
19/01/2016	Yes	19	14	516	<16	344	100 ± 15	--	--	<50	--	--
02/02/2016	Yes	18	14	594	<16	396	266 ± 32	--	--	<50	78 ± 19	<50
23/02/2016	Yes	19	21	546	<16	364	233 ± 28	--	--	<50	<50	--
01/03/2016	Yes	18.5	7	498	--	332	147 ± 6	--	--	180 ± 33	<50	<50
21/03/2016	No	NA	14	NA	ND	NA	123 ± 19	--	--	--	--	--
22/03/2016	Yes	21	21	627	--	418	113 ± 17	--	--	--	--	--
24/05/2016	Yes	19	7	579	<16	386	--	--	--	--	--	--
20/06/2016	Yes	17	7	546	--	364	--	--	--	--	<50	--
17/10/2017	Yes	19	21	543	--	362	<50	--	--	--	--	--
31/10/2017	Yes	17.5	14	600	--	400	--	<50	--	<50	<50	--
28/11/2017	Yes	17.5	28	609	--	406	<50	--	--	<50	<50	<50
30/01/2018	No	NA	63	NA	ND	NA	141 ± 71	--	--	--	--	--
17/04/2018	Yes	18	140	558	ND	372	92 ± 21	<50	--	<50	--	<50
Median Standard deviation		18.5 1.78	17.5 32.03	558 41.28	0 4.10	372 27.52	100 78.37	0 10.06	0 0.00	25 79.34	0 31.15	0 14.74

--, Not detected; NA, non applicable; ND, not determined.

Regarding RBC contamination by irinotecan, 12 out of 19 samples were contaminated by irinotecan, corresponding to 63%. Irinotecan was quantified in 4 (21%) out of 19 RBC samples with a minimum of 114 pg/mL, a maximum of 257 pg/mL. Irinotecan was detected in eight out of 19 samples (42%). Fewer irinotecan-contaminated samples in RBC samples were found than irinotecan-contaminated plasma samples. However, the concentration was higher in some RBC samples than in the plasma samples (257 vs. 100 pg/mL; 241 vs. 103 pg/mL; 180 vs. 147 pg/mL). Nine samples out of 19 (47%) were contaminated with SN-38. SN-38 was quantified on 2 (78 pg/mL, 127 pg/mL) out of 19 samples (10%) and was detected in seven out of 19 samples, corresponding to 36%. Seven samples were contaminated with APC at concentrations near the limit of quantification.

A plasmatic irinotecan contamination (n=2) was found (92; 200 pg/mL) after the first HIPEC procedure following a long period without a HIPEC procedure (two months). Moreover, plasmatic irinotecan contamination (n=3) was also found after holidays (103; 141 pg/mL).

Regarding platinum compound contamination in the plasma, seven out of 19 samples were contaminated.

## Discussion

Of the nine published biomonitoring studies carried out in HIPEC, six are based on urine samples with the limitations of urine samples previously mentioned [27]. For these six studies, the results are quantifiable in two studies. Two studies were being carried out on the basis of blood samples, one of which is based on only five plasma samples with values below the quantification limit of 1 μg/L [33].

The benefit of the investigated irinotecan dose in this study compared to the other antineoplastic drugs was that irinotecan was present only in the hospital environment, whereas platinum could be found as a pollutant in the natural environment. The amount of irinotecan measured in the blood samples is only limited to irinotecan and its determined metabolites, whereas for platinum the amount corresponds to all molecules containing platinum. The different published results showed inconsistent contamination with platinum compounds, which could be explained by the limit of quantification of the analysis implemented. Even if this study shows blood contamination with two different antineoplastic drugs assessed in two different laboratories, further studies should be performed in other surgeons, other operating theater caregivers, other centers and different antineoplastic drugs used during HIPEC such as mitomycine C to confirm these results considering the inconsistent contamination found in the literature [27]. Toxicological studies have also to be led to assess effects at the concentration of AD found.

Another aspect of this observational study was to evaluate the contamination by an active anticancer metabolite: SN-38. Another study found irinotecan and SN-38 contamination in both plasma and urine for one cleaner and one preparatory in an Italian hospital but out of a context of HIPEC [34].

By comparing irinotecan and platinum results, irinotecan seems to be a better biomonitoring indicator than platinum compounds. The evaluation of blood contamination by antineoplastic drugs used during HIPEC should be generalized by biomonitoring surgeons. There was a good perception of surgeons to this invasive technique of blood sampling. It was perceived as a complementary tool for surgeons for carrying on working on safe handling of antineoplastic drugs and protective equipment. This biomonitoring should be more relevant than complete blood count with differential and reticulocyte count currently recommended and performed [35], [36]. This biomonitoring should also be extended to the different antineoplastic drugs used during HIPEC procedures thanks to analytical progress.

According to the concentration of irinotecan, SN-38 and APC in RBC, we could theorize that RBC could be assimilated to a reservoir model. However, it is not clear to us as to why we found no concentration of irinotecan (except in 12 out of 19 samples, 63%), SN-38 (except in nine out of 19 samples, 47%) and APC (except in seven out of 19 samples, 36%) in almost all RBC samples. According to the literature and with our understanding of irinotecan metabolism, these three molecules enter RBCs passively and are not degraded by RBC enzymes. SN-38 strongly binds blood cells, whereas irinotecan binding to blood cells is negligible [37].

In 2018, a study performed by the French National Research and Safety Institute for the Prevention of Occupational Accidents and Diseases (INRS) showed that the hospital environment was contaminated by anticancer drugs: computers, door-handles and work surfaces [38]. These results could support and explain the positive blood samples from the surgeon after a long period of inactivity and from the fellow surgeon before practicing HIPEC.

For limitations of the study, there is no link established between the dose manipulation and the amount of contamination and we could not determine the way of exposure to anticancer drugs during, before and after HIPEC. By comparing the data of this study and the INRS study, it is evident that the idea was supported by the INRS study [38].

This case study dealt with one surgeon’s blood contamination because surgeons are the closest to antineoplastic drugs and in direct contact with them. It would have been interesting to assess the contamination of other professionals in the operating room. We were able, as complementary data to sample a resident who accompanies twice the surgeon. The resident was contaminated before assisting in any HIPEC sessions by irinotecan and platinum compounds assesses by two different laboratories (one value of plasmatic irinotecan between limits of detection and quantification). This raises the hypothesis of hospital contamination outside the operating room. Contamination doesn’t seem to be limited to HIPEC operating room. We can notice that this resident previously worked in an oncology care unit during her previous internship before participating to HIPEC. After HIPEC, contamination of the resident’s sample, comprised between limits of detection and quantification, were observed for plasmatic irinotecan and for RBC irinotecan, SN-38 and APC. The “background noise” of the contamination should be assessed out of all HIPEC.

To improve the observation period, we can also record a video of the surgery to establish a link between the surgeon’s actions and the surgeon’s blood contamination by anticancer drugs. This could help future studies focus on understanding healthcare workers’ blood contamination risk; however this will not directly prevent contamination. To complete this blood contamination assessment, we can also perform surface samples to look for the source of contamination both in the operating room and ward environment even if it does not prejudge the systematic passage to exposed healthcare professionals [27]. Furthermore, from these videos, simulation training sessions could be developed to improve the surgeons’ practice during surgery concerning safety. Likewise, this practice could be extended to the rest of the operating room staff including cleaning operators who are insufficiently protected and taken into account [39], [40].

It should also be interesting to assess if the knowledge of systemic contamination with antineoplastic drugs had an impact on the perception and knowledge of the risk associated with antineoplastic drugs handling during HIPEC and above all on the adherence to handling practice guidelines.

In conclusion, this study shows, for the first time, blood contamination by irinotecan, two of its major metabolites including active SN-38 and platinum compounds both in plasma and RBC, in a surgeon performing HIPEC. Monitoring blood contamination by anticancer drugs of all healthcare workers and finding ways to prevent it is an international challenge to all involved in both surgical and medical practices. In the future, studies in this area of interest can be conducted to improve the safety of the hospital work environment and the welfare of healthcare workers. In the future studies, the different possible sources of contamination should be explored in the theater, ward and hospital environment.
